# Identifying subgroup of severe community-acquired pneumonia based on clinical metagenomics, a multicenter retrospective cohort study

**DOI:** 10.3389/fcimb.2024.1516620

**Published:** 2025-01-07

**Authors:** Mingqiang Wang, Yue Jin, Wenxiao Zhang, Ling Ye, Huanzhang Shao

**Affiliations:** ^1^ Department of Critical Care Medicine, Xinxiang Medical University, Henan Provincial People’s Hospital, Zhengzhou, China; ^2^ Department of Critical Care Medicine, Henan Key Laboratory for Critical Care Medicine, Zhengzhou Key Laboratory for Critical Care Medicine, Henan Provincial People’s Hospital; Zhengzhou University People’s Hospital, Henan University People’s Hospital, Zhengzhou, China

**Keywords:** sCAP, community-acquired pneumonia, clinical metagenomics, latent class analysis, corticosteroid

## Abstract

**Objective:**

Severe community-acquired pneumonia (sCAP) is one of the major diseases within the ICU. We hypothesize that subtyping sCAP based on simple inflammatory markers, organ dysfunction, and clinical metagenomics results is feasible.

**Method:**

In this study, we retrospectively enrolled immunocompetent sCAP patients requiring invasive mechanical ventilation, who underwent clinical metagenomics from 17 medical centers. We collected data on potentially pathogenic species reported by clinical metagenomics and clinical information for all patients. Latent class analysis (LCA) was applied to routine clinical parameters such as gender, age, white blood cell (WBC), lymphocytes, C-reactive protein (CRP), and Procalcitonin (PCT), identifying two optimally fitting models.

**Results:**

A total of 569 patients were enrolled. Compared to class B, class A was characterized by a younger age, higher CRP and PCT levels, and a higher incidence of coagulation dysfunction, liver failure, circulatory failure, and renal failure. However, the mortality rates were similar between the two groups. In class A, more cases of *Streptococcus* spp. and fewer cases of HSV-1 and *Candida* spp. were detected. Among the patients in the two phenotypes, 48.7% and 57.5% received corticosteroid treatment, respectively. In the class A, corticosteroid treatment was not associated with patient mortality (unadjusted hazard ratio (HR)=0.988; 95% confidence interval (CI), 0.634–1.541; p=0.959). In contrast, in the class B group, the use of corticosteroids was associated with a reduced mortality rate (adjusted HR=0.719; 95% CI, 0.525–0.986; p=0.04). Additional analysis showed that in class B, methylprednisolone was associated with reduced mortality (adjusted HR=0.61; 95% CI, 0.44–0.86; p=0.005), while dexamethasone was not associated with mortality (adjusted HR=1.4; 95% CI, 0.89–2.22; p=0.148). In addition, after dose conversion, the results showed that higher doses of corticosteroids in class B were associated with increased mortality (adjusted HR=1.01; 95% CI, 1.00–1.01; p=0.005).

**Conclusion:**

We identified two classes based on clinical metagenomics and clinical features. Class B exhibited a better response to corticosteroid compared to class A. The rapid identification of these phenotypes could facilitate the screening of sCAP patients responsive to corticosteroid in future prospective clinical trials.

## Introduction

Severe community-acquired pneumonia (sCAP), a common cause of mortality within the ICU, exhibits a remarkably high incidence and mortality rate (Dequin et al., 2023). The benefits of corticosteroids in sCAP patients have become increasingly evident; however, there remain unanswered questions (Snijders et al., 2010; Rochwerg, 2022; Dequin et al., 2023; Wu et al., 2023; Xu et al., 2023). Specifically, it is unclear whether all sCAP patients can benefit from corticosteroids. Conducting subgroup analysis on sCAP may lay the groundwork for developing precise medical strategies for its management. Currently, patient stratification in sCAP is based on clinical features and host responses (Antcliffe et al., 2019; Pereverzeva et al., 2022). Despite numerous randomized controlled trials reporting on the pathogens of sCAP, there is limited research categorizing them based on the causative microorganisms (Snijders et al., 2010; Blum et al., 2015; Wirz et al., 2016; Dequin et al., 2023). Therefore, in this multicenter, retrospective, proof-of-concept study, we hypothesize that subgroups characterized by different lung microbiota profiles will exhibit varying responses to corticosteroids. We retrospectively collected clinical data from 1,897 patients who underwent clinical metagenomics across 17 medical centers and differentiated potential categories using latent class analysis (LCA).

## Method

### Patients and data collection

This multicenter retrospective cohort study was conducted in adult ICUs in 17 medical centers in China. A total of 1,897 patients with severe pneumonia and underwent clinical metagenomics testing of BALF from January 2019 to June 2023 were enrolled as described before (Jiang et al., 2024; Wei et al., 2024b). The study has been approved by the ethics committees of Henan Provincial People’s Hospital and other participating hospitals. As a retrospective study, informed consent was waived. All research was performed in accordance with relevant guidelines. Research involving human research participants followed the Declaration of Helsinki. If clinical metagenomics was performed multiple times, the results obtained from the first instance were included in our analysis. The definition of severe pneumonia was as previous (Mandell et al., 2007). Exclusion criteria were as follows: 1. age <18 years; 2. loss to follow-up or abandoning treatment within 28 days after ICU admission; 3. pneumonia classified as CAP; 4. clinical metagenomics performed more than 7 days after ICU admission; 5. immunosuppression, defined as previously described (Huang et al., 2023a; Huang et al., 2023b); and 6. not received invasive mechanical ventilation. The reason for selecting patients who underwent clinical metagenomics within 7 days was that we suggested that for sCAP patients, the likelihood of secondary infections would significantly increase beyond 7 days, meaning that the mNGS results might no longer reflect the original pathogens. The definition for corticosteroid treatment is continuous administration for at least 2 days after hospital admission. The data related to demographics and medical history were collected from the patients’ medical charts. Therefore, there was no need to exclude any parameters included in the analysis. Missing data were imputed using multiple imputation.

### Clinical metagenomics assay and selection of candidate microbiota

The DNA clinical metagenomics testing laboratory was certified by either the College of American Pathologists or the External Quality Assessment program of the Chinese National Health Commission (Huang et al., 2021; Huang et al., 2023a). The detailed information is provided in **Supplementary Material**. In this study, clinical metagenomics reported over 100 microbial species. We chose to include species detected in >10% of the patients as model parameters (considering genus level for bacteria and fungi, and species level for viruses). We deemed this approach practical, avoiding an unnecessary increase in the model’s complexity. The microorganisms that we reported are unbiased. Any microbial information detected through the NGS workflow and consistent with our analytical pipeline is included in the results.

### Identification of subclasses

Latent class analysis (LCA) was conducted to identify the subclasses of patients with R-package depmixS4 as described (Filippini et al., 2022; Soussi et al., 2022). The process was performed independent of clinical outcomes. Age, sex, lymphocyte, neutrophil, and CRP, PCT, and candidate microbiota were included in the LCA. Highly correlated variables (Spearman correlation coefficient > 0.7 and p-value < 0.05) were excluded from the LCA. The optimal model was evaluated by Bayesian Information Criterion (BIC), Akaike Information Criterion (AIC), and Lo–Mendell–Rubin likelihood ratio test (LMR-LRT) and the number of patients in the classes.

### Statistical analysis

Categorical data were expressed with frequency and rate or proportion. Student’s t-test, Wilcoxon, and Kruskal–Wallis tests were used to compare continuous data. Counting data were compared with chi-squared test, cross-table test, or Fisher’s exact test. Survival analysis was conducted with Cox proportional hazards models. Since, in our study, the definition for corticosteroid use is continuous administration for 2 days after hospital admission, we included only patients with a survival time of 2 days or more in the Cox model (as all patients in the corticosteroid groups had a survival time >2 days). A two-sided p < 0.05 was considered statistically significant. The analyses were performed with R through the R-studio.

## Results

**Figure 1:**
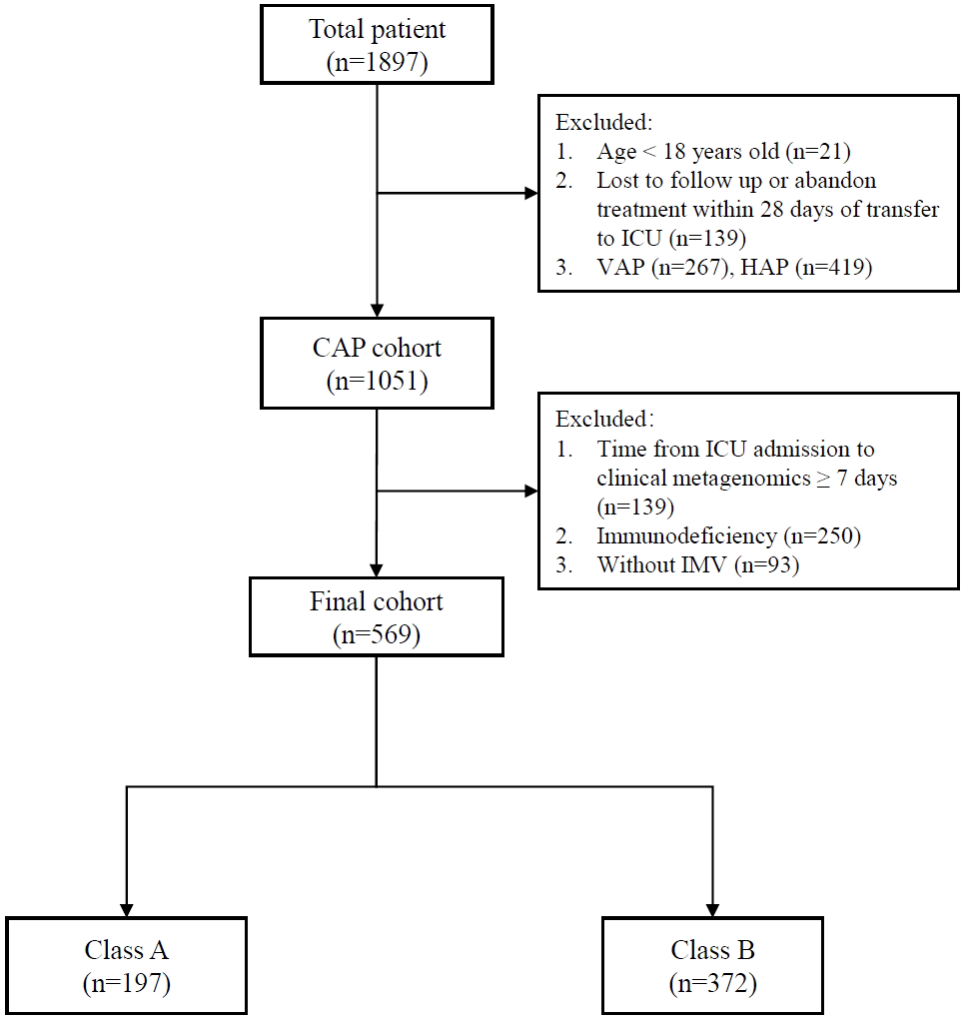
Flow chat.

**Figure 2:**
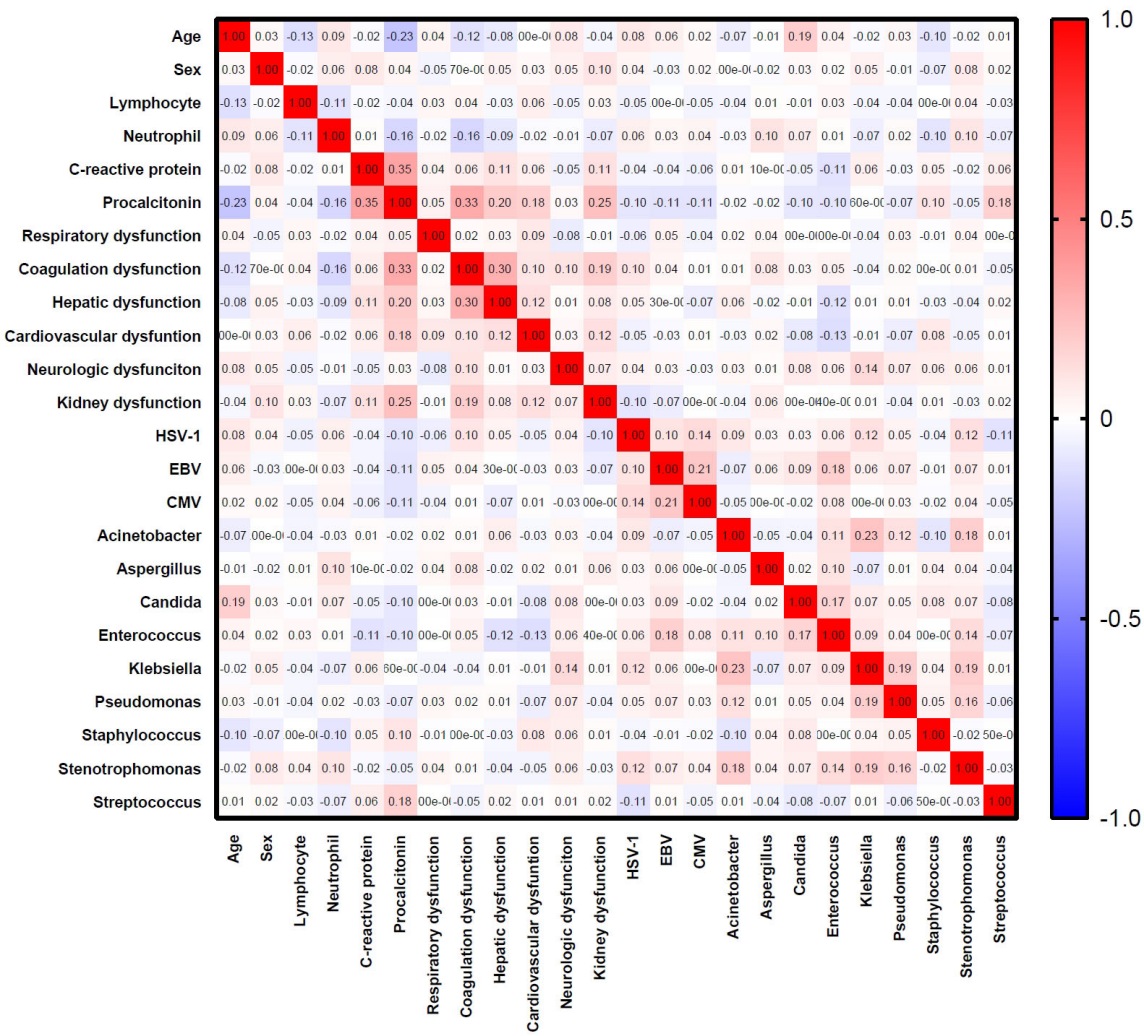
Heatmap of correlation between selected variables for phenotyping.

A total of 569 patients were included in the analysis (**Figure 1**). Variables including age, sex, lymphocyte, neutrophil, C-reactive protein, procalcitonin, HSV-1, CMV, EBV, *Aspergillus* spp., *Candida* spp., *Acinetobacter* spp., *Klebsiella* spp., *Enterococcus* spp., *Pseudomonas* spp., *Staphylococcus* spp., *Stenotrophomonas* spp., *Streptococcus* spp., and organ dysfunction were included (**Table 1**). The correlation coefficients between the variables were all <0.7 (**Figure 2**). Although the Bayesian Information Criterion (BIC) gradually decreased with an increase in the number of classes, the two-class model was considered the optimal model (**Table 2**). This decision was made because in the three to five class range, there were classes with <5% of the total population.

**Table 1:** Clinical characteristics of the patients.

	Total (n=569)	LCA results		
		Class A (n=197)	Class B (n=372)	p-value
Variables included in LCA analysis				
Age, year, median (IQR)	68.0 (58.0–77.0)	64.0 (52.0–73.0)	71.0 (60.0–78.0)	**<0.001**
Male, n (%)	395 (69.4)	137 (69.5)	258 (69.4)	0.963
Lymphocyte, 10^9^/L	0.6 (0.3–0.9)	0.5 (0.3–0.9)	0.6 (0.3–0.9)	**0.001**
Neutrophil, 10^9^/L	10.3 (5.9–15.1)	8.6 (4.0–15.5)	10.7 (7.2–15.0)	0.369
C-reactive protein, mg/L	104.3 (47.8–180.4)	158.9 (92.7–250.6)	80.8 (37.7–139.7)	**<0.001**
Procalcitonin, ng/mL	1.3 (0.3–9.6)	24.4 (8.5–70.7)	0.5 (0.2–1.4)	**<0.001**
Organ dysfunction, n (%)				
Respiratory	508 (89.3)	179 (90.9)	329 (88.4)	0.374
Coagulation	185 (32.5)	101 (51.3)	84 (22.6)	**<0.001**
Hepatic	84 (14.8)	48 (24.4)	36 (9.7)	**<0.001**
Cardiovascular	315 (55.4)	133 (67.5)	182 (48.9)	**<0.001**
Neurologic	229 (40.2)	76 (38.6)	153 (41.1)	0.555
Kidney	131 (23.0)	79 (40.1)	52 (14.0)	**<0.001**
Microorganisms reported by clinical metagenomics, n (%)				
HSV-1	117 (20.6)	25 (12.7)	92 (24.7)	**<0.001**
CMV	73 (12.8)	18 (9.1)	55 (14.8)	0.055
EBV	78 (13.7)	22 (11.2)	56 (15.1)	0.200
*Aspergillus* spp.	86 (15.1)	29 (14.7)	57 (15.3)	0.849
*Candida* spp.	174 (30.6)	46 (23.4)	128 (34.4)	**0.006**
*Acinetobacter* spp.	146 (25.7)	44 (22.3)	102 (27.4)	0.186
*Klebsiella* spp.	178 (31.3)	63 (32.0)	115 (30.9)	0.794
*Enterococcus* spp.	73 (12.8)	19 (9.6)	54 (14.5)	0.098
*Pseudomonas* spp.	75 (13.2)	23 (11.7)	52 (14.0)	0.440
*Staphylococcus* spp.	61 (10.7)	25 (12.7)	36 (9.7)	0.269
*Stenotrophomonas* spp.	67 (11.8)	18 (9.1)	49 (13.2)	0.155
*Streptococcus* spp.	64 (11.2)	35 (17.8)	29 (7.8)	**<0.001**
Variables not included in LCA analysis				
Comorbidities, n (%)				
Diabetes mellitus	151 (26.5)	53 (26.9)	98 (26.3)	0.886
Myocardial infarction	33 (5.8)	10 (5.1)	23 (6.2)	0.591
Chronic pulmonary disease	122 (21.4)	28 (14.2)	94 (25.3)	**0.002**
Liver disease	27 (4.7)	9 (4.6)	18 (4.8)	0.885
Chronic kidney disease	57 (10.0)	28 (14.2)	29 (7.8)	**0.015**
Solid tumor	64 (11.2)	19 (9.6)	45 (12.1)	0.378
Cerebrovascular disease	110 (19.3)	34 (17.3)	76 (20.4)	0.362
SOFA, median (IQR)	8.0 (5.0–10.0)	10.0 (7.0–13.0)	7.0 (5.0–9.0)	**<0.001**
Corticosteroid treatment	310 (54.5)	96 (48.7)	214 (57.5)	**0.045**
Type of corticosteroid				0.144
methylprednisolone	262 (46)	83 (42.1)	179 (48.1)	
dexamethasone	44 (7.7)	11 (5.6)	33 (8.9)	
hydrocortisone	4 (0.7)	2 (1)	2 (0.5)	
Dose	40 (40, 80)	40 (40, 80)	40 (40, 80)	0.102
Usage days	7 (5, 12)	6.5 (4, 10)	7 (5, 13)	0.041
Viral pneumonia				0.411
influenza A	36 (6.3)	15 (7.6)	21 (5.6)	
COVID-19	137 (24.1)	45 (22.8)	92 (24.7)	
Other	1 (0.2)	1 (0.5)	0	
Bloodstream infection	138 (24.3)	59 (29.9)	79 (21.2)	**0.021**
28day-mortality, n (%)	265 (46.6)	96 (48.7)	169 (45.4)	0.453

IQR, Interquartile range; BALF, bronchoalveolar lavage fluid; HSV-1, Herpes simplex Virus 1; CMV, Cytomegalovirus; EBV, Epstein–Barr virus.

Organ dysfunction is represented by a SOFA score of 2 or higher for each component.. p<0.05.

Bold values: p<0.05.

**Table 2:** Fit statistics for latent class models.

Classes	BIC	AIC	LMR-LRT p-value	Entropy	Number of class
1	18,786.3	18,660.4			None
2	15,833.6	15,577.3	<0.0001	0.946	A: 197; B: 372
3	15,063	14,676.4	<0.0001	0.925	A: 329; B: 223: C: 17
4	14,748.4	14,231.5	<0.0001	0.88	A: 19; B: 227; C: 190; D: 133
5	14,887.9	14,240.7	p: =0.5002	0.878	None

(BIC), Bayesian Information Criterion; (AIC), Akaike Information Criterion; and (LMR-LRT), Lo–Mendell–Rubin likelihood ratio test. p<0.05.

The two classes were defined as class A and class B, and the clinical and microbial characteristics between the total population and the two groups are shown in **Table 1**. Compared to class B patients, class A patients were characterized by a lower age (64 vs. 71, p<0.001), lower lymphocyte count (0.5 vs. 0.6, p=0.001), higher CRP (158.9 vs. 80.8, p<0.001), and higher PCT (24.4 vs. 0.5, p<0.001). In terms of comorbidities, class A patients had less chronic pulmonary disease (14.2% vs. 25.3%, p=0.002) and more chronic kidney disease (14.2% vs. 7.8%, p=0.015). Meanwhile, the baseline occurrence rates of respiratory failure and neurological dysfunction were similar between the two groups. However, class A had higher rates of coagulation dysfunction (51.3% vs. 22.6%, p<0.001), liver dysfunction (24.4% vs. 9.7%, p<0.001), circulatory dysfunction (67.5% vs. 48.9%, p<0.001), and kidney dysfunction (40.2% vs. 14.0%, p<0.001). Class A patients had a higher SOFA score (10.0 vs. 7.0, p<0.001). In class A patients, the detection rate of *Candida* spp. (23.4% vs. 34.4%, p=0.006) and the detection rate of HSV-1 (12.7% vs. 24.7%, p<0.001) were lower, while the detection rate of *Streptococcus* spp. was higher (17.8% vs. 7.8%, p<0.001). The types and doses of corticosteroids used in the two groups showed no statistically significant differences. However, the duration of corticosteroid use was longer in the class B group (7 vs. 6.5, p=0.041), which may be attributed to the lower mortality rate in this group. Additionally, we reported the proportion of viral pneumonia and the incidence of bloodstream infections in the two patient groups. As shown, the types of viral pneumonia were similar between the two groups, but the class B group had a lower incidence of bloodstream infections (21.2% vs. 29.9%, p=0.021). Meanwhile, we compared the mortality risk of patients using corticosteroids. Those patients using corticosteroids in class B had lower risk of mortality, but there was no statistically significant difference (HR=0.665; 95% CI, 0.414–1.067; p=0.091).

Later, we examined the responses of these two groups to corticosteroids (**Table 3**). Multivariate Cox models were separately conducted in both groups. It can be observed that in class A, the use of corticosteroids was not associated with patient prognosis (unadjusted HR=0.988; 95% CI, 0.634–1.541; p=0.959). However, in class B, the use of corticosteroids was correlated with a lower 28-day mortality rate (adjusted HR=0.719; 95% CI, 0.525–0.986; p=0.04). Additionally, we reported the effects of corticosteroid use types and doses on patient mortality. The results show that in class A, methylprednisolone was not associated with patient mortality (p=0.566), whereas dexamethasone was associated with increased mortality (p=0.006). In class B, methylprednisolone was associated with reduced mortality (p=0.005), while dexamethasone showed no association with mortality (p=0.148). Additionally, after dose conversion, the results indicate that higher doses of corticosteroids in class B were associated with increased mortality (p=0.005). However, it is important to note that this is a *post-hoc* analysis, and further prospective studies are needed for validation. Additionally, we provide the number of patients using corticosteroids in each medical center (**Supplementary Table S4**).

**Table 3:** Cox model of mortality at day 28 in two groups.

	Class A univariate		Class A Multivariate		Class B univariate		Class B Multivariate	
	HR (95%CI)	p-value	HR (95%CI)	p-value	HR (95%CI)	p-value	HR (95%CI)	p-value
Corticosteroid treatment	0.988 (0.634–1.541)	0.959	/	/	0.792 (0.579–1.083)	0.145	0.719 (0.525–0.986)	**0.040**
Age	1.015 (1.002–1.029)	**0.029**	1.020 (1.006–1.034)	**0.005**	1.021 (1.009–1.033)	**<0.001**	1.020 (1.008–1.032)	**0.001**
Male	0.788 (0.492–1.261)	0.321	/	/	1.023 (0.731–1.445)	0.875	/	/
Lymphocyte	1.035 (0.973–1.101)	0.277	/	/	0.964 (0.681–1.365)	0.837	/	/
Neutrophil	0.999 (0.979–1.019)	0.897	/	/	1.010 (0.987–1.034)	0.379	/	/
C-reactive protein	0.999 (0.997–1.001)	0.240	/	/	1.001 (0.999–1.003)	0.315	/	/
Procalcitonin	1.000 (0.994–1.006)	0.930	/	/	1.141 (1.002–1.300)	**0.047**	/	/
SOFA score	1.130 (1.067–1.196)	**<0.001**	1.144 (1.082–1.210)	**<0.001**	1.141 (1.090–1.194)	**<0.001**	1.145 (1.092–1.200)	**<0.001**

Adjusted for corticosteroid treatment and all of other parameters in unadjusted model with a p-value < 0.15. Stepwise model selection was adopted.

SOFA, sequential organ failure assessment.

Since, in our study, the definition for corticosteroid use is continuous administration for 2 days after hospital admission, we included only patients with a survival time of 2 days or more in the Cox model (as all patients in the corticosteroid groups had a survival time >2 days). p<0.05.

Bold values: p<0.05.

## Discussion

In this study, we included the most critically ill patients with severe pneumonia, nearly all of whom required mechanical ventilation. The average SOFA score of these patients was 8, and the mortality rate was extraordinarily high, approaching 50%. In such cases, ICU clinicians often administer single-dose corticosteroids in response to hemodynamic instability. However, this does not represent continuous corticosteroid therapy in this patient population. Therefore, we excluded patients who received only single-dose corticosteroids to clearly identify those who underwent prolonged corticosteroid treatment. Considering that an immunocompromised state can lead to a more complex and variable landscape of pathogenic microorganisms in the lungs and could diminish the effectiveness of stratification, patients with immunosuppression were excluded. Our research indicates that by combining simple inflammatory markers, including lymphocytes, neutrophils, CRP, PCT, along with organ dysfunction, and correlating them with suspected pathogenic microorganisms in the lungs, we can effectively subgroup sCAP patients. In summary, in our study, mechanically ventilated sCAP patients included had the highest mortality risk, with both groups demonstrating increased organ dysfunction compared to previous research and notably high mortality rates for both Group A and Group B (Meduri et al., 2022; Dequin et al., 2023).

The systemic use of glucocorticoids has been recommended for critically ill patients with CAP, but current evidence remains controversial (Meduri et al., 2022; Dequin et al., 2023). Pathogens detected in the lungs, such as influenza and coronavirus disease 2019 (COVID-19), have shown varying responses to corticosteroids (File and Ramirez, 2023; Martin-Loeches et al., 2023). Identifying subgroups responsive to corticosteroids through pathogen stratification is one direction for future research. In our study, we found that the use of corticosteroids did not improve patient outcomes for class A patients, while for class B patients, corticosteroids reduced the risk of death. Combining these patients’ significantly elevated PCT and more frequent organ dysfunction, we have reason to believe that Group A patients may experience more systemic bacterial dissemination, not just lung infection. Additional data shows that class A patients experienced more frequent bloodstream infection. As *Streptococcus* spp. is more frequently detected in this group, it is also one of the main causative microorganisms for secondary meningitis in CAP (Martin-Cerezuela et al., 2023). Our study may weaken the argument for the benefits of corticosteroids in patients with *Streptococcus* spp. infection and significantly elevated PCT.

Our conclusions support previous findings from two clinical trials on corticosteroid treatment for CAP, suggesting that CAP caused by *Streptococcus pneumoniae* infection may have a poorer response to corticosteroids (Snijders et al., 2010; Wirz et al., 2016). In one clinical trial, CAP caused by *Streptococcus pneumoniae* exhibited significantly higher levels of PCT and CRP compared to pneumonia not caused by *Streptococcus pneumoniae*, which aligns with our cohort data (Wirz et al., 2016). The rationale for using corticosteroids in sCAP patients is to counteract tissue damage caused by pathogen-induced host inflammatory responses (Dequin et al., 2023). However, for class A patients, the reasons for pathogen-induced tissue damage may include not only host inflammatory responses but also widespread dissemination and direct invasion by the pathogen, which could be one of the reasons for the significant increase in PCT. While many studies have grouped sCAP based on host responses, most have not discussed or lacked the capability to identify the pathogens causing sCAP (Pereverzeva et al., 2022). Our study demonstrates that LCA grouping based on clinical metagenomics can distinguish between groups responsive and non-responsive to hormones.

What sets our study apart from other subgroup-distinguishing studies is that all the patients we included are sCAP, not sepsis (unlike the VANISH study, where only approximately 50% of patients had lung infections, which would increase heterogeneity in the results) (Antcliffe et al., 2019). At the same time, we obtained information on lung microbial, which is lacking in other clinical trials. Meanwhile, the patients we enrolled have better homogeneity. We excluded immunosuppressive patients because their lung microbiota detection is more complex. Meanwhile, we included sCAP patients who require mechanical ventilation, who have a higher mortality rate. For these patients, is the microbial infection or host response more crucial? We cannot answer this question, but it does not prevent us from raising this question and taking such a small step in this field.

While it is possible that physicians may identify potential corticosteroid-benefiting patients during the diagnostic and therapeutic process, it is important to note that our LCA analysis was conducted in a blinded manner for the clinicians. They were unaware of whether the patients they treated were classified as class A or class B. In our study, for the class A group, although clinicians chose to administer corticosteroids to some patients, this clearly did not impact the mortality rate of this group. This still underscores the point that class B patients are more likely to benefit from corticosteroid therapy compared to class A patients. However, cautious interpretation is necessary, and confirmation through well-designed prospective trials is still needed. In our retrospective study, we adjusted for confounding factors widely believed to be associated with death, such as age and SOFA score, but there were still potential confounding factors that were not adjusted for, such as other concurrent medications. At the same time, our study included sCAP patients with the highest mortality rate, which also limited our conclusion from being extended to milder sCAP patients. In addition, although corticosteroids are widely used in patients with acute respiratory distress syndrome (ARDS) and COVID-19, it is undeniable that there may be heterogeneity in the use of corticosteroids among centers in multicenter studies. Finally, our study only included DNA clinical metagenomic data, which may overlook the impact of RNA viruses on grouping results. Future research directions may involve incorporating the absolute load of detected bacteria and viruses and other inflammatory markers including IL-6 into models to achieve more accurate results. An ongoing prospective study that we are conducting may provide further insights into this issue (Wei et al., 2024a).

## Conclusion

In this study, we observed that utilizing clinical metagenomics and straightforward clinical features allowed us to subgroup immunocompetent sCAP patients requiring mechanical ventilation into two categories. Group A, characterized by elevated detection of *Streptococcus* spp. and higher inflammatory markers, exhibited a poorer response to corticosteroids.

## Data Availability

The original contributions presented in the study are included in the article/**Supplementary Material**. Further inquiries can be directed to the corresponding authors.
