# Supplementary material for: Identifying subgroup of severe community-acquired pneumonia based on clinical metagenomics, a multicenter retrospective cohort study
Source: Front Cell Infect Microbiol. 2025 Jan 7;14:1516620. doi: 10.3389/fcimb.2024.1516620 (PMC11753243; doi:10.3389/fcimb.2024.1516620)
Supplement: Supplementary file 1 [file Table1.docx]

**Supplement method**

mNGS Workflow

DNA-based mNGS testing for BALF samples of each patient was performed in the clinical laboratory. BALF DNA was extracted from 1 mL of the sample using the QIAamp® UCP Pathogen DNA Kit (catalog number: 50214, Qiagen, Duesseldorf, Germany) according to the manufacturer’s instructions. Human DNA was removed using 1U Benzonase (Sigma) and 0.5% Tween 20 (Sigma) and incubated at 37°C for 5 min. The extracted DNA was then quantified using a Qubit dsDNA HS Assay Kit (catalog number: Q32854, Invitrogen, Carlsbad, CA, USA). Subsequently, the quantified unique DNA fragments (named UMSI) were spiked for each sample as an identity and internal control, which were PCR products of Oryza sativa of 400 to 600 bp in length. Thirty microliters of the eluate were used to generate libraries using the Nextera DNA Flex kit (Illumina, San Diego, CA, USA) according to the manufacturer’s instructions. Library pools were then loaded onto the Illumina Nextseq CN500 sequencer for 50 cycles of single-end sequencing (SE-50), generating approximately 20 million reads for each library. For negative controls, Balf samples were also prepared from healthy donors in parallel with each batch using the same protocol, and sterile deionized water was extracted alongside the specimens to serve as non-template controls.

Low-quality reads, adapter contamination, duplicate reads, and low-complexity reads were removed by fastp (version 0.20.0) with default parameters. Human sequence data were excluded by mapping to a human reference genome (hg38) using Burrows-Wheeler Aligner (version 0.7.17). The remaining sequencing data were aligned to an in-house microbial database for microbial identification with SNAP v1.0 beta. The company designed a set of criteria similar to the National Center for Biotechnology Information (NCBI) criteria for selecting representative assemblies for microorganisms (bacteria, viruses, fungi, protozoa, and other multicellular eukaryotic pathogens) from the NCBI Nucleotide and Genome databases (https://www.ncbi.nlm.nih.gov/assembly/help/anomnotrefseq/). Pathogen lists were selected according to three references:

1) Johns Hopkins ABX Guide (https://www.hopkinsguides.com/hopkins/index/Johns_Hopkins_ABX_Guide/Pathogens),

2) Manual of Clinical Microbiology (https://www.clinmicronow.org/doi/book/10.1128/9781683670438.MCM),

3) clinical case reports or research articles published in peer-reviewed journals. The final database consisted of approximately 13,000 genomes.

Microbial reads were aligned to the database with SNAP v1.0beta.18 (https://arxiv.org/abs/1111.5572). *P. jirovecii*-positive detection results were defined as the coverage of one or more non-overlapping regions on the genome. Virus-positive detection results (DNA viruses) were defined as the coverage of three or more non-overlapping regions on the genome. For an important human pathogen that is parasitic in cells, it is difficult to extract nucleic acids (e.g., Rickettsia app.) was considered positive when SMRN > 1. Other positive detection was reported for a given species or genus if the reads per million (RPM) ratio (RPM-r) was ≥ 5, where RPM-r was defined as the RPM sample/RPMNC (i.e., the RPM corresponding to a given species or genus in the clinical sample divided by the RPM in the negative control (NC)). In addition, to minimize cross-species misalignments among closely related microorganisms, we penalized (reduced) the RPM of microorganisms sharing a genus or family designation if the species or genus appeared in non-template controls. A penalty of 5% was used for species.

Table S1. Cox model of mortality at day 28 in patients with Corticosteroid treatment

|  | Class A univariate | |  | Class A Multivariate | |
| --- | --- | --- | --- | --- | --- |
|  | HR (95%CI) | P value |  | HR (95%CI) | P value |
| Class B | 0.634 (0.380 - 1.057) | 0.080 |  | 0.665 (0.414 – 1.067) | 0.091 |
| Age | 1.024 (1.011 - 1.037) | **<0.001** |  | 1.023 (1.010 - 1.036) | **<0.001** |
| Male | 0.814 (0.557 - 1.191) | 0.289 |  | / | / |
| Lymphocyte | 1.101 (1.064 - 1.140) | **<0.001** |  | 1.101 (1.065 - 1.139) | **<0.001** |
| Neutrophil | 1.000 (0.981 - 1.019) | 0.964 |  | / | / |
| C-reactive protein | 0.999 (0.997 - 1.001) | 0.546 |  | / | / |
| Procalcitonin | 0.992 (0.982 - 1.002) | 0.100 |  | 1.009 (0.982 – 1.001) | 0.067 |
| SOFA score | 1.149 (1.092 - 1.208) | **<0.001** |  | 1.145 (1.090 – 1.204) | **<0.001** |

Adjusted for all of other parameters in unadjusted model with a P value less than 0.15. Stepwise model selection was adopted.

Abbreviation: SOFA, sequential organ failure assessment.

Table S2. Cox model of mortality at day 28 in two group.

|  | Class A univariate | | Class A Multivariate | | Class B univariate | | Class B Multivariate | |
| --- | --- | --- | --- | --- | --- | --- | --- | --- |
|  | HR (95%CI) | P | HR (95%CI) | P | HR (95%CI) | P | HR (95%CI) | P |
| Corticosteroid treatment |  |  |  |  |  |  |  |  |
| methylprednisolone | 0.91 (0.55 – 1.50) | 0.709 | 0.87 (0.53 – 1.41) | 0.566 | 0.63 (0.44 – 0.89) | **<0.001** | 0.61 (0.44 – 0.86) | **0.005** |
| dexamethasone | 2.79 (1.36 – 5.72) | **0.005** | 2.68 (1.32 – 5.43) | **0.006** | 1.43 (0.90 – 2.29) | 0.131 | 1.40 (0.89 – 2.22) | 0.148 |
| hydrocortisone | None | None | None | None | None | None | None | None |

Adjusted for Corticosteroid treatment and all of other parameters in unadjusted model with a P value less than 0.15. Stepwise model selection was adopted.

Abbreviation: SOFA, sequential organ failure assessment.

Since, in our study, the definition for corticosteroid use is continuous administration for 2 days after hospital admission, we included only patients with a survival time of 2 days or more in the Cox model (as all patients in the corticosteroid groups had a survival time greater than 2 days).

Table S3. Cox model of mortality at day 28 in two group.

|  | Class A univariate | | Class A Multivariate | | Class B univariate | | Class B Multivariate | |
| --- | --- | --- | --- | --- | --- | --- | --- | --- |
|  | HR (95%CI) | P | HR (95%CI) | P | HR (95%CI) | P | HR (95%CI) | P |
| Dose | 1.00 (1.00 – 1.00) | 0.235 | / | / | 1.01 (1.00 – 1.01) | **0.005** | 1.01 (1.00 – 1.01) | **0.005** |

Adjusted for dose of corticosteroid and all of other parameters in unadjusted model with a P value less than 0.15. Stepwise model selection was adopted.

Abbreviation: SOFA, sequential organ failure assessment.

Since, in our study, the definition for corticosteroid use is continuous administration for 2 days after hospital admission, we included only patients with a survival time of 2 days or more in the Cox model (as all patients in the corticosteroid groups had a survival time greater than 2 days).

Convert the dose of 5mg dexamethasone to 40mg methylprednisolone.

Table S4. The number of patients using corticosteroids in each medical center

| Unit | Percentage of Corticosteroid usage |
| --- | --- |
| Unit 1 | 15/26 |
| Unit 2 | 5/7 |
| Unit 3 | 19/94 |
| Unit 4 | 15/26 |
| Unit 5 | 1/1 |
| Unit 6 | 2/5 |
| Unit 7 | 1/1 |
| Unit 8 | 4/4 |
| Unit 9 | 62/91 |
| Unit 10 | 7/19 |
| Unit 11 | 23/35 |
| Unit 12 | 41/49 |
| Unit 13 | 3/7 |
| Unit 14 | 51/119 |
| Unit 15 | 3/4 |
| Unit 16 | 58/81 |

Note: Two of the medical centers belong to the same medical group and cannot be distinguished, so they are classified as one.
